# Disclosing the True Critical Point of Fluids Confined in Nanopores

**DOI:** 10.1021/acs.langmuir.5c05643

**Published:** 2026-02-18

**Authors:** Ephraim Kakra Owusu-Banahene, Sugata P. Tan, Morteza Dejam, Hertanto Adidharma

**Affiliations:** † Department of Energy and Petroleum Engineering, 4416 University of Wyoming, Laramie, Wyoming 82071, United States; ‡ 53469 Planetary Science Institute, Tucson, Arizona 85719, United States; § Department of Chemical and Biomedical Engineering, University of Wyoming, Laramie, Wyoming 82071, United States

## Abstract

Two methods for determining the pore critical point (PCP), i.e., the critical point of confined fluid, are currently available in the literature. The better-known method is based on the analysis of data obtained from adsorption isotherms, while the newer method analyzes the heat of capillary condensation data obtained from differential scanning calorimetry (DSC). For the first time, these two methods are implemented on the same confined system for comparison to explore which method provides the true PCP. While the heat released at the PCP derived from adsorption isotherms is measurably nonzero, implying that a phase transition still occurs in the pores, no heat is released at the PCP derived from the DSC measurements, which indicates the true critical point of the first-order phase transition. With this true PCP, the Peng–Robinson cubic equation of state (EOS), without any modification, is shown to accurately represent the whole capillary-condensation curve of confined pure fluids with small and large molecules as well as a confined mixture.

## Graphical abstract

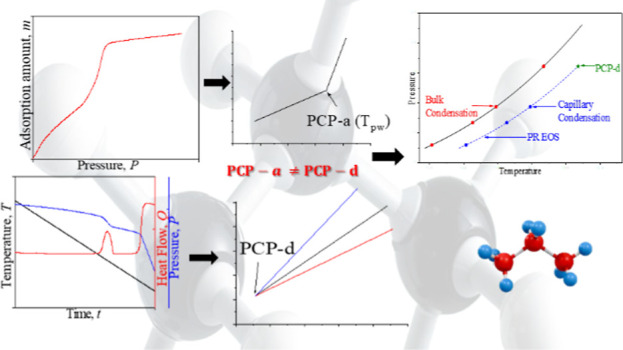

## Introduction

The phase transition of fluids confined in nanopores, known as capillary condensation, is very important both in science and in its practical applications, such as membrane separation, adsorption/desorption gas separation, gas/oil recovery from shale formation, catalysis, etc. The critical point of a confined fluid, termed the pore critical point (PCP), is another important piece of information for describing the complete phase behavior of the confined fluid. Both the capillary-condensation pressure and the PCP of confined pure fluids have been found to be lower than those of their bulk counterparts.

There are two methods for determining PCP in the literature. The better-known method is based on the analysis of data obtained from adsorption isotherms.^1–10^ The PCP in this phenomenological approach is determined by plotting the inverse slope of the inflection point (or simply, the midpoint) of the increasing adsorption step associated with capillary condensation of many isotherms against their respective temperature.^1^ The newer method, referred to as the three-line approach,^11^ is based on the vanishing heat released during capillary condensation as the system condition approaches PCP. The heat released during capillary condensation, as measured by differential scanning calorimetry (DSC), exhibits a linear relationship with both the inverse of the absolute condensation temperature (1/*T*) and the natural logarithm of the condensation pressure (ln­(*P*)), leading to the well-known Clapeyron equation.

There has been evidence that the PCPs obtained from these two methods are different, where the one derived from adsorption isotherms is notably lower than that from DSC, as pointed out in Adidharma and Tan,^12^ but to date, there are no experimental and modeling efforts to determine which one is the true PCP. To resolve this conflicting issue, we implement both methods in this work for determining the PCP of propane in 4.1 nm MCM-41. This is the first time that the two methods are compared using an identical fluid-adsorbent system. The adsorption experiments are performed to obtain the adsorption isotherms and their capillary-condensation pressures, while the DSC experiments are also performed to obtain capillary condensation conditions and the corresponding released heat. We then present our comparison analysis and propose new insights into the resulting critical points and corresponding phase transitions of confined fluids.

With the measured PCP, any equation of state (EOS) with critical points as parameters, such as the cubic EOS, whether modified or not, and an explicit EOS that is based on the modified van der Waals partition function,^13^ may use the PCP data in calculating the capillary-condensation curve. For the definition of explicit EOS, please refer to our review paper.^12^

Since fluids confined in nanopores undergo first-order phase transition, as also happens with the bulk fluids in free space, the common cubic EOS, without modification, should also be effective to work with confined fluids if the PCP is known, in the same way as bulk fluids with known critical points. The immediate use of cubic EOS in this manner makes the approach different from many previous works that apply a modification using the Laplace equation of surface tension to include the capillary pressure. The success of the immediate use was demonstrated using the Peng–Robinson (PR) EOS in a recent work by Kallehbasti and Sakhaee-Pour^14^ with small molecules, but not yet with larger ones and mixtures.

In this work, the PR EOS is applied to small and larger molecules, i.e., methane, ethane, carbon dioxide, and propane in various nanopores as well as a methane/propane mixture in SBA-15 nanopores. The PCP of propane in 4.1 nm MCM-41 is obtained in this work, while those of others were obtained in our previous works.^11,15–17^

## Experimental Section

Experiments are performed in this work to generate adsorption isotherms and measure the capillary condensation conditions of propane in MCM-41, the results of which are used to determine the PCP of the system.

### Materials

The propane gas is of research grade with a purity of 99.99% and is purchased from Airgas Specialty Gases. To obtain MCM-41 with the larger pore size needed in this work, we perform a pseudomorphic transformation of 8.1 nm SBA-15. The SBA-15 nanoporous media and hexadecyltrimethylammonium (CTAB) used are purchased from Sigma-Aldrich, sodium hydroxide pellet ACS reagent (NaOH) was obtained from Oakwood Chemical, and 200 proof ethanol is obtained from Decon Laboratories, Inc. All chemicals are used as received.

### Pseudomorphic Transformation of SBA-15

The pseudomorphic transformation of SBA-15 is prepared according to the procedure of Zucchetto et al.^18^ An amount of 106.2 mg of SBA-15 is mixed with 61.3 mg of CTAB and 17.6 mg of NaOH. To this mixture, 2 mL of H_2_O is added and stirred for 30 min in a soda lime glass tube with a screw cap. The mixture is then heated in an oven at 100 °C for 6 h. The transformed silica material is obtained by filtration, washed with 200 mL of water, and then heated to 550 °C at a heating rate of 2 °C/min, after which calcination at 550 °C is performed in air for 12 h.

The SBA-15 and transformed SBA-15 (MCM-41) nanoporous media are characterized using BET (Brunauer–Emmett–Teller) analysis. The equipment and specific procedure for sample characterization can be referred to from our previous work.^19^

Figure 1 shows the nitrogen adsorption and desorption isotherms of transformed SBA-15 (MCM-41) at 77 K and the NLDFT pore size distributions of SBA-15 and transformed SBA-15 (MCM-41). According to the IUPAC Technical Report,^20^ the isotherm of N_2_ gas in SBA-15 exhibits type IV (a) with an H1-type hysteresis loop typical of mesoporous adsorbents with larger mesopores (not shown), while the transformed SBA-15 (MCM-41) exhibits type IV (b) without a distinct hysteresis loop, which is typical of mesopores of smaller diameters. The modal diameter of the pores obtained from NLDFT is adopted in this work and corresponded to the peak of the pore size distribution in Figure 1. Based on Figure 1, the nanopores used do not seem to show any microporosity. The main properties of the adsorbents are summarized in Table 1.

**Figure 1:**
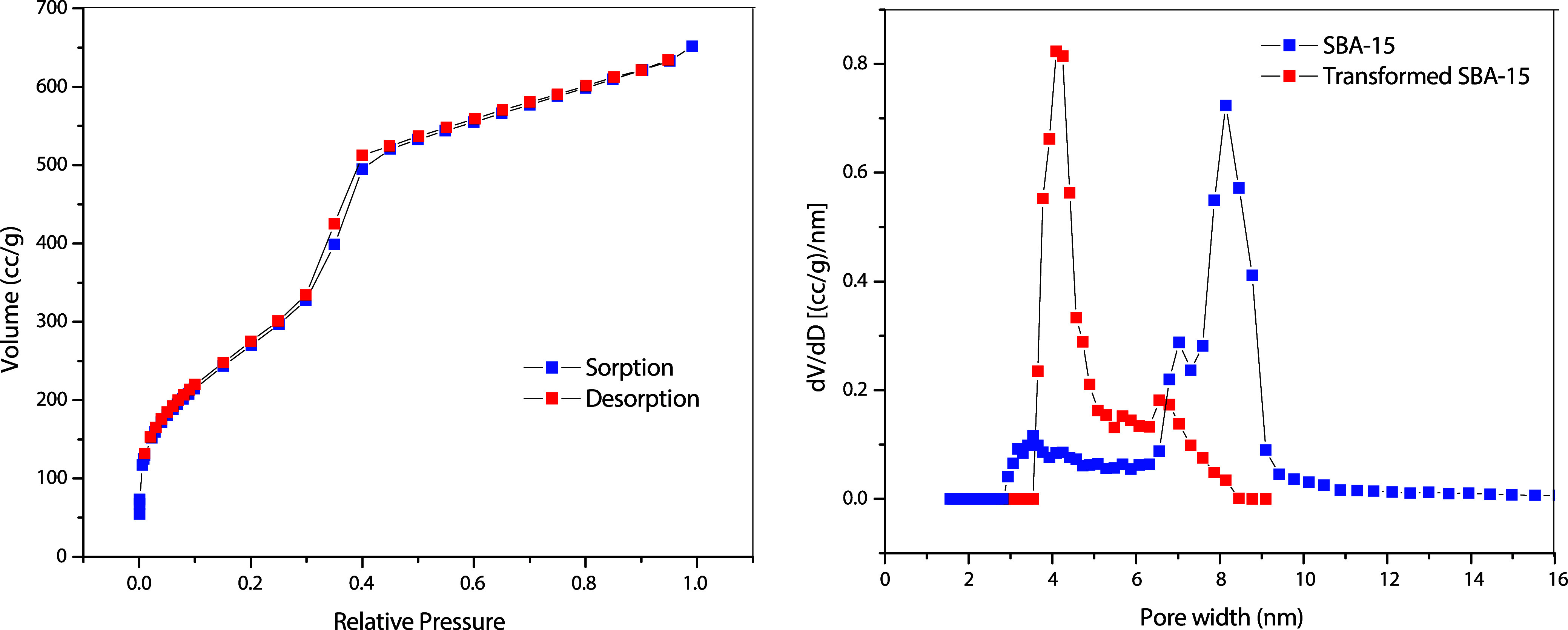
(Left): Nitrogen adsorption and desorption isotherms of transformed SBA-15 (MCM-41) at 77 K; (right): NLDFT pore size distributions of SBA-15 and transformed SBA-15 (MCM-41).

**Table 1:** Properties of Adsorbents Derived from the Nitrogen Adsorption Isotherm at 77 K

sample	*S*_BET_, m^2^/g	*V*_total_, cm^3^/g	*D*_BJH_, nm	*D*_NLDFT_, nm
SBA-15	772	1.469	6.6	8.15
transformed SBA-15 (MCM-41)	974.3	1.240	3.1	4.09

### Apparatus

The HIDEN ISOCHEMA IGA-001 gravimetric gas sorption analyzer is used to obtain the adsorption isotherms. The analyzer can operate over a wide temperature range, depending on the cooling system used, with a pressure limit of 20 bar. A cabinet encloses three essential modules of the analyzer, i.e., a computer module that acquires signals from different sensors and transmits control commands from the accompanying software, a balance chamber, and a gas handling module. Detailed information about the system can be found in the work of Yang et al.^21^ A PRESTO dynamic temperature control system manufactured by JULABO USA, Inc., is used in conjunction with the IGA system to adjust and regulate the sample temperature to the desired value for isotherm measurement and can operate over a temperature range of −80 °C to +250 °C, a control stability ranging from ±0.01 to ±0.05 °C, a cooling capacity of 1.2 kW, and a pumping capacity of 38 L/min. The circulating coolant used is a thermal P90 dodecamethylpentasiloxane, also purchased from JULABO USA, Inc.

In this work, the high-pressure SETARAM μDSC VII instrument is used to measure the first set of capillary condensation conditions at a high temperature range for PCP determination. The μDSC has a pressure limit of 400 bar, an operating temperature range of −45 to 120 °C, and a resolution of 0.04 μW. This DSC is equipped with an advanced Peltier with an auxiliary cooling circulator. For more details, readers are referred to Qiu et al.^19^ The high-pressure, low-temperature SETARAM BT 2.15 DSC instrument is used to measure the second set of capillary condensation conditions at a lower temperature range, which are used to analyze the trend of heat released around the PCP obtained from isotherm analysis as a function of temperature. It is equipped with a tank for liquid nitrogen, which is used during the DSC cooling procedure. The DSC can operate under a temperature range of −196 to 200 °C, a pressure limit of 600 bar, with a scanning rate of 0.01–1 °C/min, a power detection limit of 2–20 μW, a temperature uncertainty of 0.5 °C, and a heat measurement uncertainty of 0.2%. For more details, readers are referred to Yang et al.^16^

### Experimental Procedures

The isotherm measurements are performed using the same procedure as in the work of Yang et al.^21^ A specific amount of the MCM-41 sample is loaded into the sample container of the IGA-001 sorption analyzer. The system is then evacuated at 120 °C for at least 8 h to remove any residual gases (e.g., air) from the adsorbent and connecting lines. Using the accompanying Isochema HIsorp software, an isotherm measurement sequence is programmed, consisting of a pretreatment stage, a stepwise pressure sequence for adsorption and desorption, and a post-treatment stage. The sequence is initiated once the cooling circulator stabilizes at the desired temperature. For the adsorption process, used to generate the adsorption isotherm, the system pressure is incrementally increased by simultaneously opening the inlet valve and closing the exhaust valve to introduce propane gas. Conversely, for the desorption process, the pressure is gradually reduced by closing the inlet valve and opening the exhaust valve. Throughout the experiment, the sample temperature, system pressure, and sample weight are automatically recorded by the software. This procedure is repeated for each isotherm measurement at different temperatures.

The general experimental procedures for using μDSC VII and BT 2.15 DSC are the same. The MCM-41 sample is placed in an oven and heated at 120 °C for 12 h. The isochoric method described in our previous work is then followed in this work.^16,19^ A small amount of the pretreated MCM-41 sample is introduced into the test vessel using a spatula. The test system is then evacuated using a vacuum pump to remove air and moisture. The temperature of the DSC system is initially set and maintained at a temperature at least 30 K above the bulk dew point temperature. Propane is then injected into the system using a syringe pump until the desired initial pressure is reached, after which the system is allowed to equilibrate for 2 h. Once equilibration has been reached, the temperature of the system is decreased at a constant cooling rate of 0.05 °C/min.

## Methods of Determining Pore Critical Points

### Adsorption Isotherm Method

Figure 2 shows a typical adsorption isotherm for a uniform mesoporous medium with a narrow range of pore size distribution, such as the MCM-41 used in this work, where a sharp increase in the adsorbed amount is observed at a short-range of pressure along an isotherm, which is the signature of capillary condensation. The step is thereby used to determine the capillary-condensation pressure.

**Figure 2:**
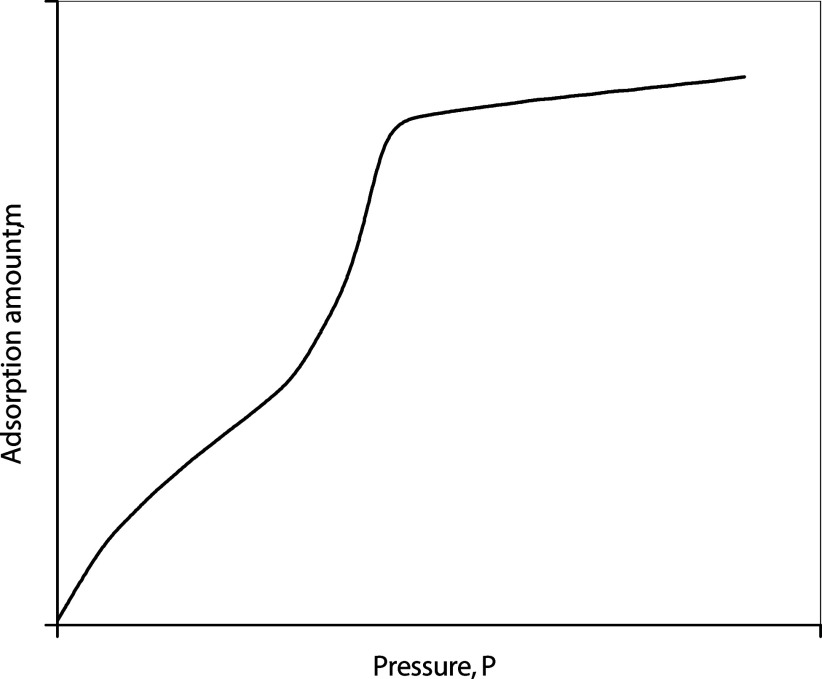
Typical adsorption isotherm with capillary condensation.

The midpoint of the step is conventionally identified as the capillary condensation condition by numerous researchers.^3,22^ In this work, a method using the inflection point is employed to determine the capillary-condensation condition from the isotherms, as it signifies the maximum condensation rate and allows for an objective determination.^4^ An isotherm is fitted by using a cubic spline, and the first derivative of the adsorbed amount with respect to pressure (*dm*/*dP*) can be easily obtained from the fitted curve. The capillary condensation pressure for a given isotherm (temperature) is identified as the maximum of the first derivative, which can be obtained either through the second derivative of the fitted isotherm curve or by fitting the first derivative to a Lorentzian function. Both methods agree well. The Lorentzian function is considered the natural shape corresponding to a first-order phase transition for a confined fluid. Therefore, the function offers an additional test for first-order phase transitions in the pores.

The inverse of the maximum value of *dm*/*dP* for every isotherm is then plotted against temperature to determine the pore critical temperature, here called as *T*_Cp‑a_. The temperature at the breakpoint or the intersection point between the “lines” of the low- and high-temperature sections is considered the *T*_Cp‑a_.^1^ The corresponding pore critical pressure (*P*_Cp‑a_), which is actually the pressure of the surrounding bulk at this PCP, is obtained by interpolating the capillary condensation conditions around *T*_Cp‑a_. This method will be demonstrated in the Results and Discussion section. The PCP derived from this method is termed PCP-a in this paper.

### Heat of Condensation Method

Figure 3 shows a typical thermogram obtained from a DSC cooling procedure for a fluid confined in a uniform mesoporous sample with a narrow range of pore size distribution. The thermogram exhibits a small, distinct exothermic peak of the heat flow during the capillary condensation and a large exothermic peak for bulk condensation. The procedure for determining the capillary condensation pressure and temperature is described in our previous works.^16,19^

**Figure 3:**
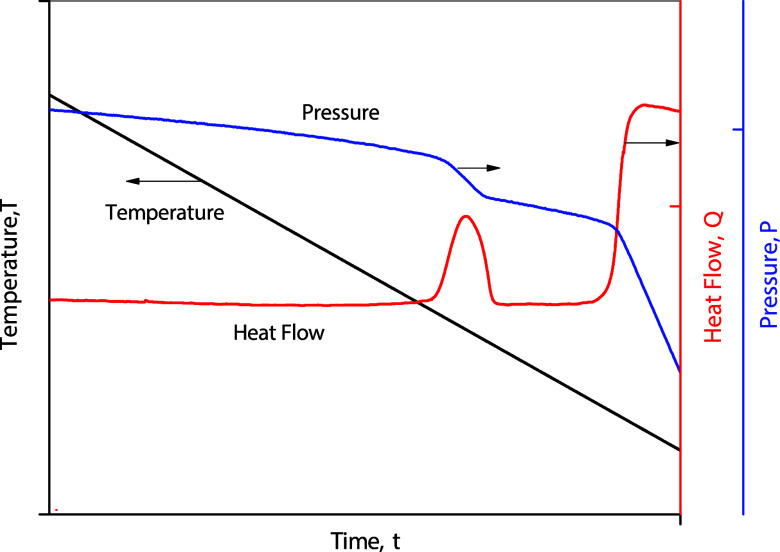
Schematic illustration of a typical thermogram obtained from a DSC cooling procedure for a system with capillary condensation.

The heat released in the phase transition is obtained from the area under the capillary condensation peak in the thermogram, which can be obtained automatically by using DSC after identifying the initial and end points of the peak on the baseline. If the reciprocal of absolute temperature and the natural logarithm of pressure during capillary condensation are plotted against the released heat, they show a linear relationship, which is thus consistent with the Clapeyron equation. The intercepts of the two plots, corresponding to zero heat, represent the reciprocal of the pore critical temperature, called here as *T*_Cp‑d_, and the logarithm of the critical pressure, called here as *P*_Cp‑d_. As the slopes of the lines are dependent on the quantity of nanoporous media utilized, three different amounts of media may be used for more accurate values of the intercepts. This method, known as the three-line approach, will be demonstrated in the Results and Discussion section. The derived PCP is termed PCP-d in this paper.

### Application of Peng–Robinson EOS

Since the PR EOS is well established and commonly used in many applications, its details can be widely found in the literature as well as college textbooks. Readers interested in reviewing the EOS are referred to a good textbook, e.g., by Smith et al.^23^ Here, we will discuss the parameters needed in the EOS to produce the saturation curve of a pure chemical species. In addition to the critical-point temperature (*T*_C_) and pressure (*P*_C_), which are experimentally measured for any individual pure chemical species, the EOS also needs another parameter known as the acentric factor (ω). It is a measure of molecular nonsphericity, thus approaching zero for spherical molecules. Pitzer et al.^24^ effectively define it as

1a $$\omega \equiv -1.0-\log\left(P^{sat}/P_{C}\right)\;at\;T=0.7\cdot T_{C}$$

where *P* ^sat^ is the saturated vapor pressure at the temperature *T* = 0.7·*T*_C_. Therefore, ω can be obtained for any fluid from *T*_C_, *P*_C_, and a single vapor-pressure measurement at *T* = 0.7·*T*_C_.

For confined pure fluids, the critical point is the PCP at (*T*_Cp_, *P*_Cp_), and the capillary condensation condition at *T* = 0.7·*T*_Cp_ is easily obtained from the experimental data used for deriving the PCP, e.g., by extrapolation along the Clapeyron line, which proves valid for confined fluids up to the PCP.^11,25^ The acentric factor of confined fluids is readily modified from eq 1a by replacing (*T*_C_, *P*_C_) with (*T*_Cp_, *P*_Cp_):

1b $$\omega_{p}\equiv -1.0-\log\left(P^{cc}/P_{Cp}\right)\;at\;T=0.7\cdot T_{Cp}$$

where *P* ^cc^ is the capillary-condensation pressure at *T* = 0.7·*T*_Cp_.

Since there is no phase-coexistence region found for confined fluid mixtures,^26^ unlike that for bulk fluid mixtures sandwiched by bubble-point and dew-point curves, the capillary-condensation curve of a confined fluid mixture behaves just like that of a pure species, i.e., it is a single curve that ends at the PCP. Therefore, the PR EOS only needs the above three parameters, *T*_Cp_, *P*_Cp_, and ω_p_, to represent the capillary-condensation curve for both pure fluids and mixtures, as shown later in the next section.

## Results and Discussion

### Adsorption–Desorption Experiments

Figure 4 shows the adsorption and desorption isotherms of propane in 4.1 nm MCM-41 nanopores at sufficiently high temperatures, where no hysteresis is observed, indicating that the temperatures investigated are above the hysteresis end point (HEP), where capillary condensation and evaporation coincide. It is worth noting that hysteresis does not affect the determination of the pore critical point because the hysteresis phenomenon always ends at the HEP, which occurs at a much lower temperature than the pore critical temperature *T*_Cp_. As expected, a sharp change in the adsorbed amount is observed for all isotherms measured, which is the signature of capillary condensation/evaporation. This sharpness suggests uniform mesopores with a narrow range of pore size distribution, as illustrated by the unimodal pore size distribution derived from the nitrogen adsorption isotherm at 77 K. As shown in Figures 5a and 5b, the Lorentzian function is applied to determine the capillary-condensation pressure for the respective isotherms, i.e., at the maximum of the function. However, as seen in Figure 5b, the determination of the inflection point using a Lorentzian function becomes more challenging as the temperature increases, because the *dm*/*dP* curve is a peak on a slanted baseline. The fitting Lorentzian curve must be rotated and shifted.

**Figure 4:**
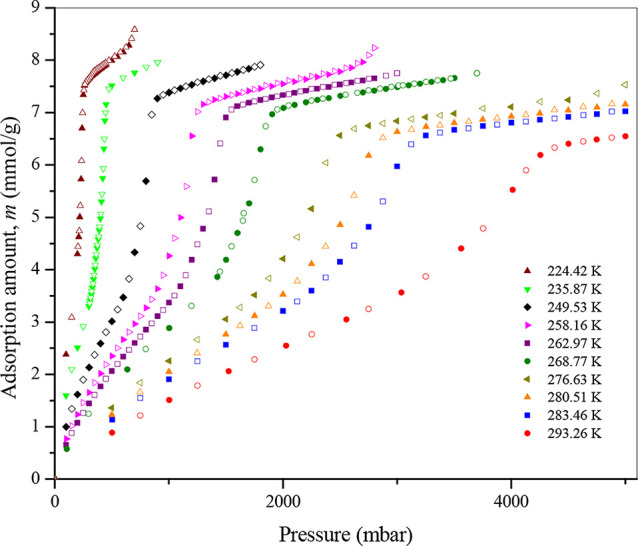
Adsorption–desorption isotherms of propane in 4.1 nm MCM-41. Filled symbols denote adsorption, and open symbols denote desorption.

**Figure 5:**
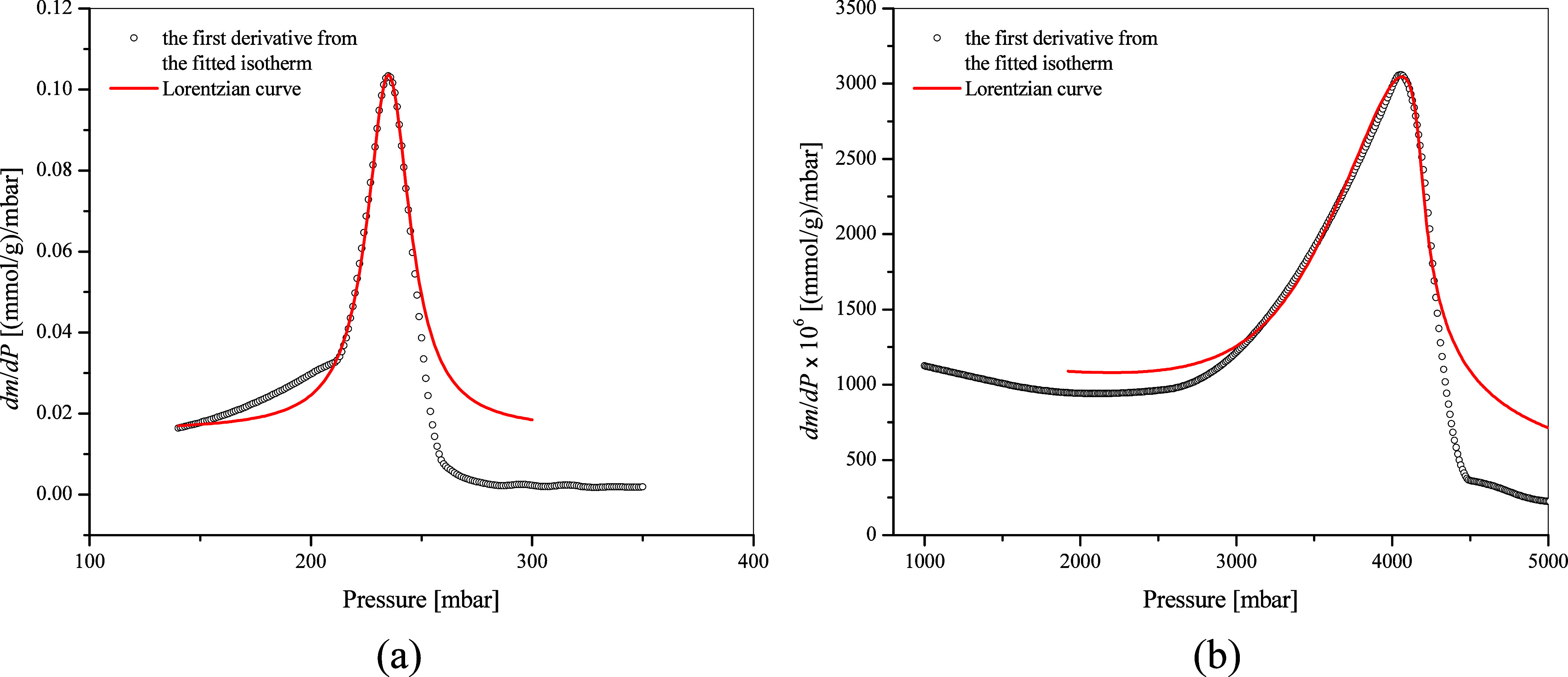
Lorentzian curve fit to identify capillary condensation pressure of propane in 4.1 nm MCM-41 (a) at 224.4 K and (b) at 293.3 K.

In Figure 6, the inverse of the maximum value of *dm*/*dP* for every isotherm is plotted against its temperature to determine *T*_Cp‑a_, which is at the breakpoint where the slope apparently changes.^1^ From our experiments, PCP-a for propane in 4.1 nm MCM-41 using this method is found to be at 268.8 K and 1.726 bar. For easier comparison, the derived capillary-condensation pressures and PCP-a are presented on the *P*–*T* diagram in Figure 7 together with those from DSC measurements presented in the next section.

**Figure 6:**
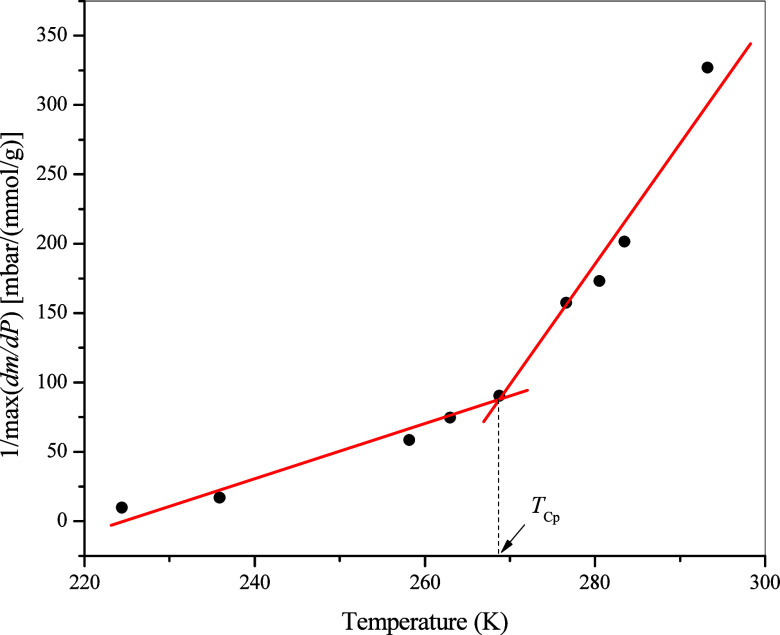
Determination of *T*_Cp‑a_ for propane in 4.1 nm MCM-4 according to Morishige et al.^1^

**Figure 7:**
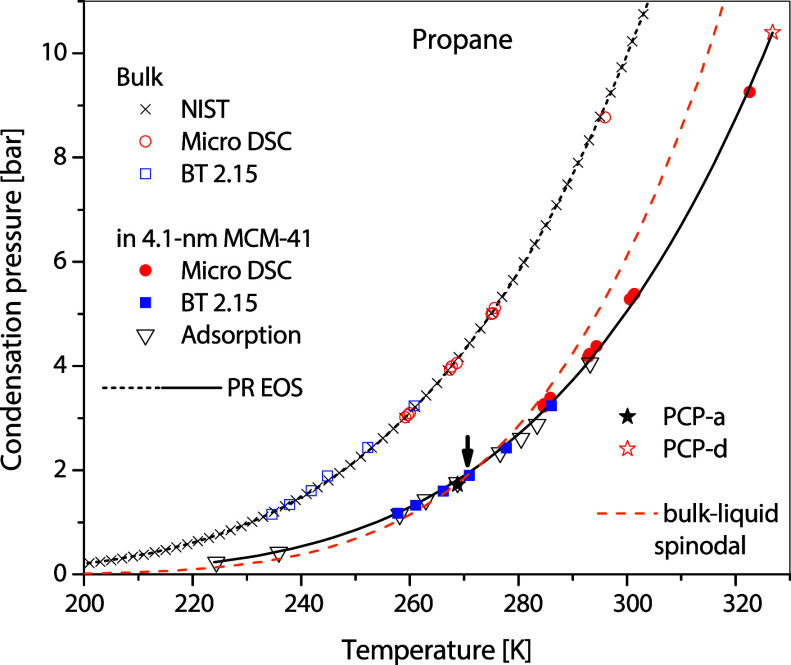
*P*–*T* diagram of C_3_H_8_ confined in 4.1 nm MCM-41 measured using adsorption isotherms and DSC. The bulk vapor pressure was measured using DSC to check the experimental accuracy. The small downward arrow is the estimate of the breakpoint in Appendix A.

### DSC Experiments

Figure 8 shows a typical thermogram of μDSC VII of propane in 4.1 nm of MCM-41. The BT 2.15 DSC has similar thermograms. The first and second sets of measured capillary condensation and bulk condensation conditions are shown in the *P*–*T* diagram in Figure 7. The bulk and capillary condensation conditions are measured in a single run, allowing the error associated with the capillary condensation measurements to be calculated. The uncertainties can be found in the Supporting Information. The first set of capillary condensation conditions, along with the corresponding released heat, is used in the three-line approach to determine PCP-d presented in Figure 9.

**Figure 8:**
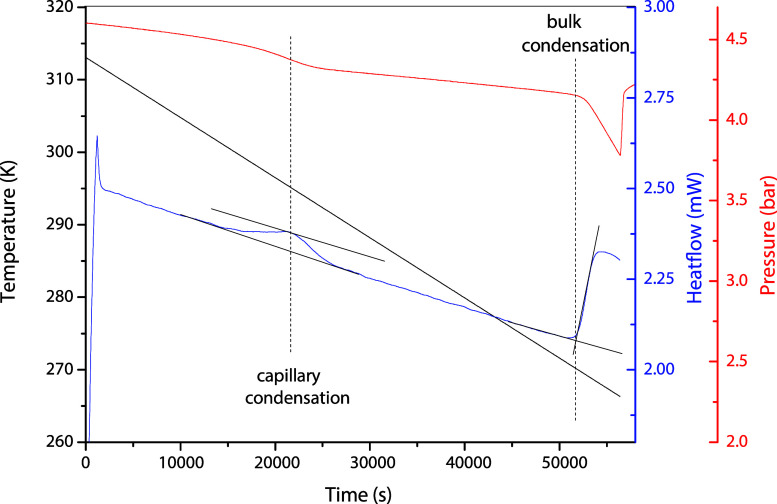
Typical DSC thermogram in the measurement of bulk and capillary condensation conditions of propane in 4.1 nm MCM-41.

**Figure 9:**
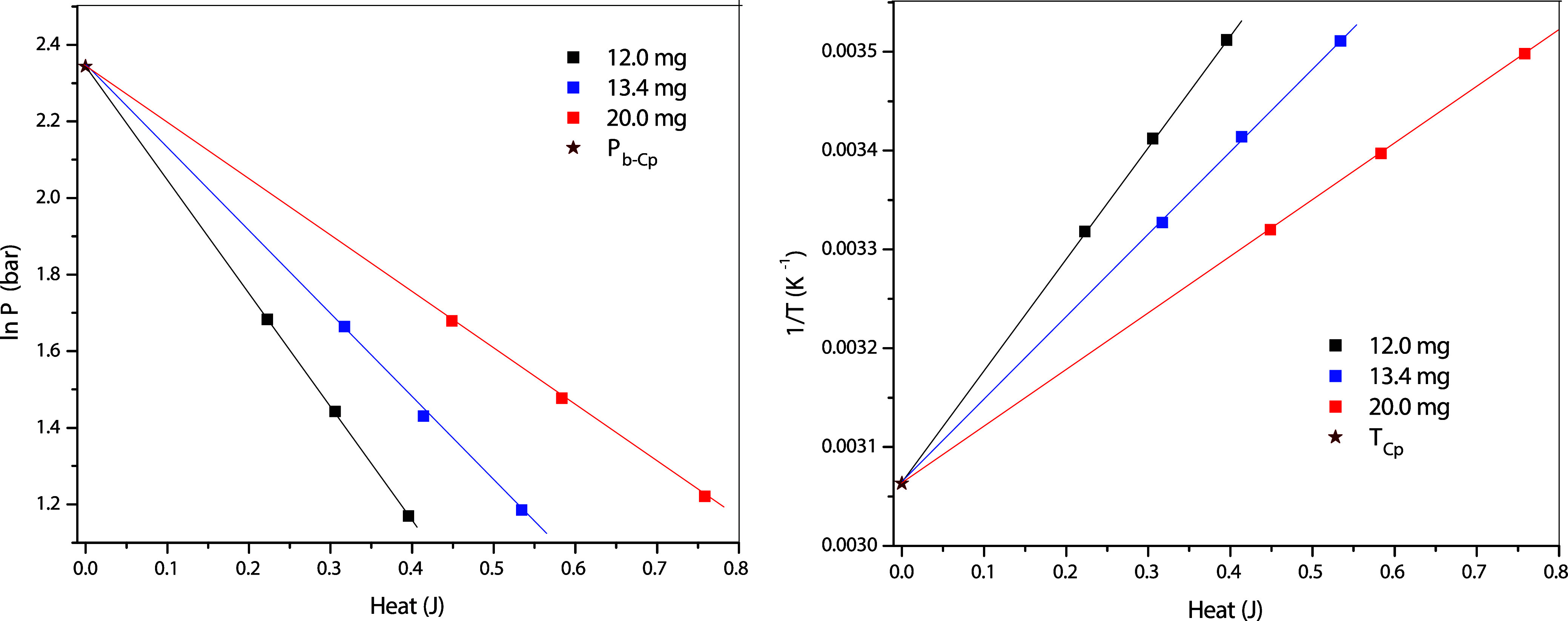
Three-line approach to obtain *P*_Cp‑d_ (left) and *T*_Cp‑d_ (right) of propane confined in 4.1 nm MCM-41. Symbols: capillary condensation data; lines: regressions; stars: the conditions at the PCP-d.

Three different adsorbent amounts, i.e., 12.0, 13.4, and 20.0 mg, are used to measure the capillary-condensation conditions for this purpose. Figure 9 shows that the plots for nanoconfined propane exhibit excellent linearity, as expected. By using this three-line approach, *T*_Cp‑d_ and *P*_Cp‑d_ of propane confined in MCM-41 are found to be 326.8_–0.9_^+0.2^ K and 10.4_–0.2_^+0.3^ bar, respectively. From the NIST Webbook, the critical point of bulk propane is at *T*_C_ = 369.9 K and *P*_C_ = 42.5 bar. The *T*_Cp‑d_ and *P*_Cp‑d_ of propane confined in 4.1 nm MCM-41 are therefore far below *T*_C_ and *P*_C_, respectively, but far above *T*_Cp‑a_ and *P*_Cp‑a_, respectively. For easier comparison, the derived capillary-condensation pressures and PCP-d are shown on the *P*–*T* diagram in Figure 7 together with those from adsorption measurements.

### Critical Point Analysis

As demonstrated above, the two methods for determining PCP result in very different locations. To analyze the two methods further, the second set of capillary condensation conditions, along with the associated heat released, is used to investigate the trend of heat released during capillary condensation as a function of temperature. Figure 10 shows this trend, which is very similar to the trend of the bulk. The vertical line in Figure 10 is *T*_Cp‑a_, where the heat released is not zero, and the DSC thermograms at temperatures above *T*_Cp‑a_ exhibit an exothermic peak, such as in Figure 8, indicating that a first-order phase transition, i.e., capillary condensation, still occurs in the pores. On the other hand, the heat released at *T*_Cp‑d_ is unmistakably zero, as also shown in Figure 10. The gradual decrease in heat released during capillary condensation with rising temperature also suggests that the first-order phase transition within the pores progresses from lower to higher temperatures, passing through PCP-a and continuing until the true critical point is ultimately reached, which turns out to be at *T*_Cp‑d_. Therefore, from this point on, we drop the subscripts “*d*” as we know that the true PCP is that derived from DSC measurements.

**Figure 10:**
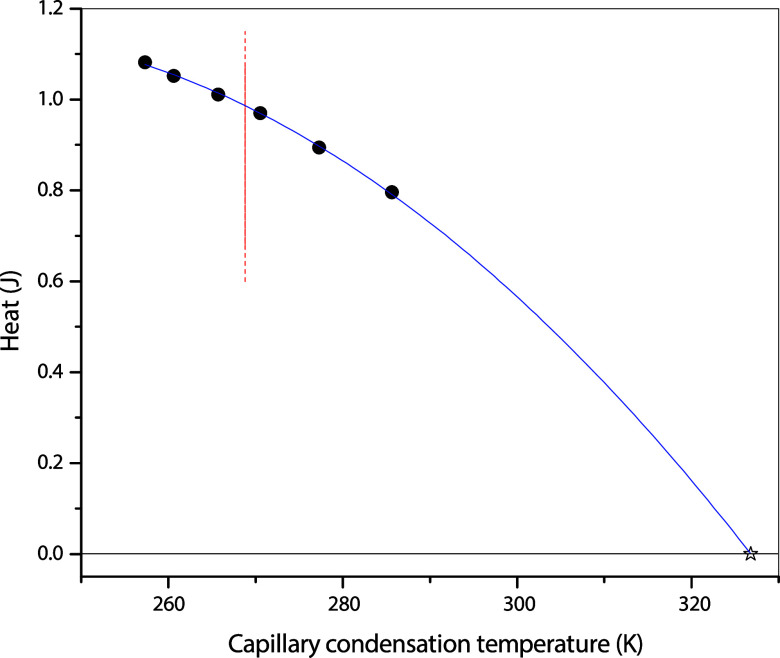
Heat released during condensation as a function of temperature. The solid curve is just a guide to the eyes. Solid circles: capillary condensation; star: *T*_Cp‑d_ from the three-line approach shown in Figure 9. The vertical line refers to *T*_Cp‑a_ derived from the adsorption-isotherm method shown in Figure 6

Nevertheless, for the adsorption isotherm method, Figure 6 does imply that there is a change in the behavior of the condensation when the temperature increases and passes through the breakpoint. As described in Tan et al.,^25^ the condensed phase in nanopores is under tension, i.e., a negative pressure, due to strong interaction with the pore walls. They showed that the breakpoint can be predicted from the spinodal conditions of the bulk liquid phase and thus related to the tensile strength of the liquid. Above this point, liquid is no longer possible to form as the adhesive force with the pore wall exceeds the maximum cohesive force that can keep the molecules staying close together as a liquid phase. This breakpoint on the *P*–*T* diagram is the intersection between the bulk-liquid spinodal curve derived using the PC-SAFT EOS in Tan et al.^25^ and the Clapeyron curve fitted to all measured capillary-condensation pressures. The bulk-liquid spinodal curve consists of bulk-vapor pressures, each of which has the same fugacity as that of the liquid spinodal at the same temperature. For propane in 4.1 nm MCM-41 in this work, the intersection is estimated in Appendix A to be at 270.56 K and 1.895 bar and is pointed to by an arrow in Figure 7, which is quite close to our experimental PCP-a, thus strongly implying that the PCP-a is the breakpoint where the negative pressure in the nanopores surpasses the liquid tensile strength.

In the bulk, a liquid phase undergoing tension higher than the liquid tensile strength cannot exist. In nanopores, at temperatures below the breakpoint, the tension exerted by the pore walls inside nanopores has not exceeded the tensile strength of the condensed phase. In other words, the cohesive interaction between fluid molecules is still larger than the adhesive interaction between the fluid molecules and pore walls. Therefore, the condensed phase partially wets the pore walls. However, at temperatures above the breakpoint, the tension exerted by the pore walls exceeds the tensile strength of the condensed phase. Since the adhesive interaction is now larger than the cohesive interaction, the condensed phase spreads and completely wets the walls. These phenomena are consistent with the theoretical study on the wetting transitions in a cylindrical pore done by Liu et al.^27^

Therefore, from now on, we better refer to this breakpoint as the pore wetting transition temperature (*T*_pw_) pointed by the down arrow in Figure 7, instead of *T*_Cp‑a_. Consequently, the well-known PCP-a measured using the adsorption method is in fact the transition condition of the confined fluid from partial wetting to complete wetting, so we can call it the pore wetting-transition point (PWTP). The wetting transition still allows confined fluid to undergo a first-order phase transition at temperatures above *T*_pw_, as evidenced by the nonzero heat release. Conventionally, the phase transition from vapor directly into a completely wetting condensed phase above *T*_pw_ has been well-known as continuous pore filling (CPF) in nanoporous-adsorption works.^12^

### Representation Using Peng–Robinson EOS

With the measured PCP handy, the capillary-condensation curve is readily calculated using PR EOS after the acentric factor is obtained, such as that demonstrated with propane in MCM-41 in Figure 7. The representations of the capillary-condensation curves of methane in MCM-41, ethane in MCM-41 and SBA-15, and CO_2_ in SBA-15 are shown in Figures 11, 12, and 13, respectively. The performance of PR EOS in representing confined CO_2_ in nanopores confirms the application of the EOS for large molecules, in addition to ethane and propane. The acentric factors used for calculating the capillary-condensation curves are listed in Appendix A, as their calculation method is discussed.

**Figure 11:**
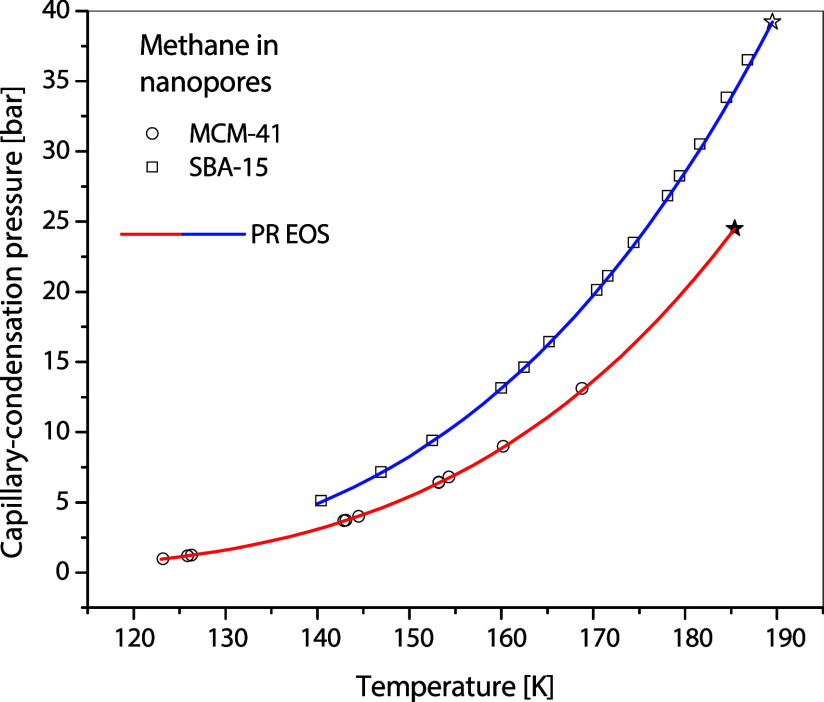
Capillary-condensation pressures of methane in 3.5 nm MCM-41 and 7 nm SBA-15,^16^ along with the corresponding curves calculated using the PR EOS.

**Figure 12:**
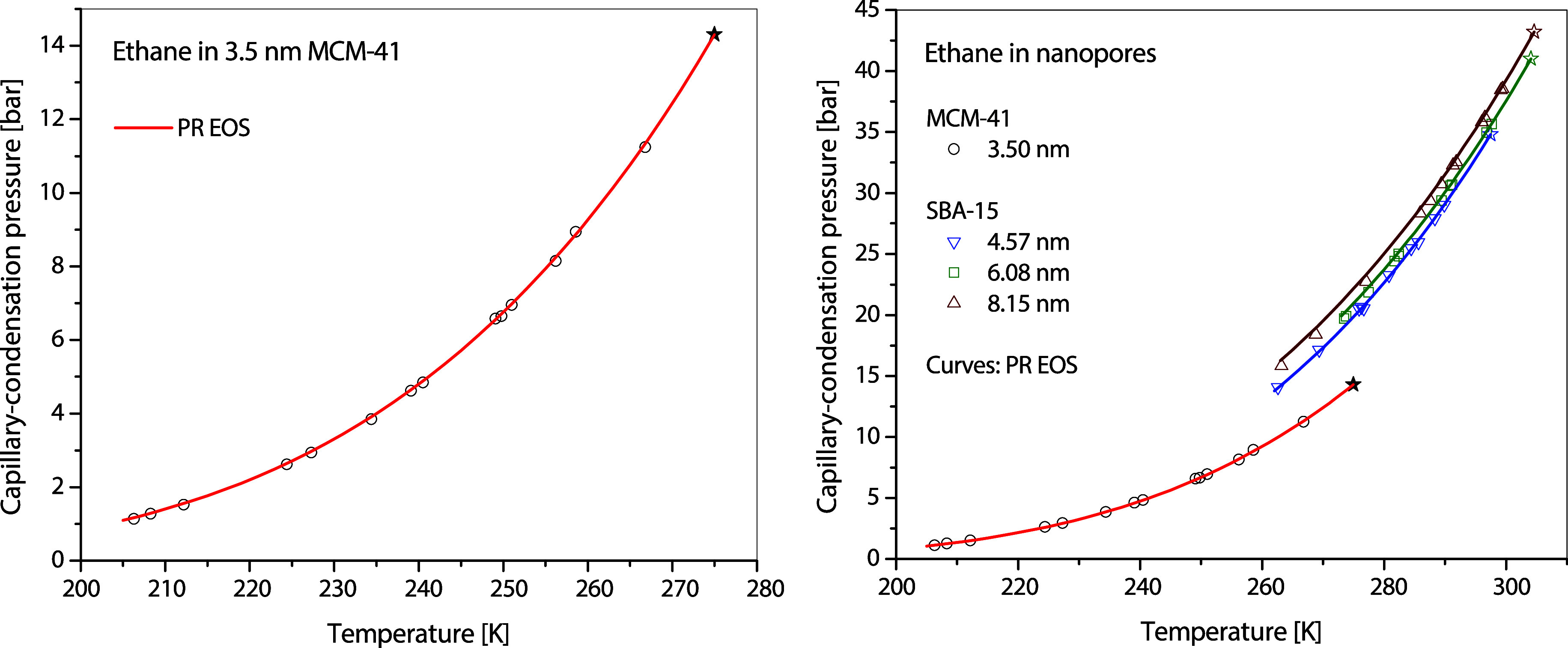
Calculated capillary condensation conditions of ethane in MCM-41 and SBA-15.

**Figure 13:**
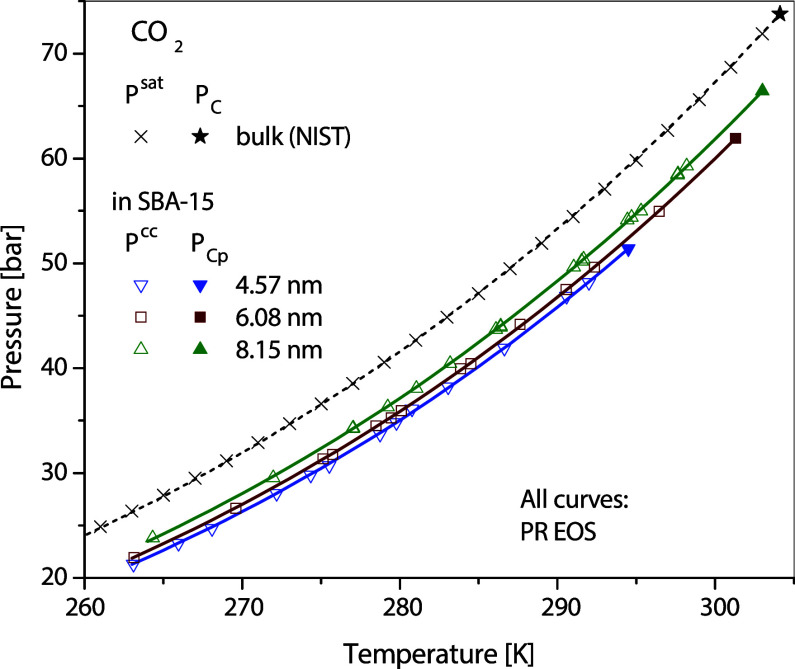
Capillary-condensation pressures of CO_2_ in SBA-15^11^ with the corresponding curves calculated using the PR EOS.

The successful demonstration of the EOS applied to confined fluids calls for future diligent efforts to make more measurements, as there is a strong indication that all the EOS parameters depend on the chemical species, as is also the case for fluids in the bulk, as well as the pore size and the porous-material type. Plots between the parameters and pore size derived from this work are given in the Supporting Information for a rough initial illustration.

Furthermore, Figure 14 even shows a strong reason for the above-needed efforts. The PR EOS can also successfully represent a confined mixture of methane/propane in MCM-41, the PCP of which has been obtained in our previous work, *T*_Cp_ = 354.6 K and *P*_Cp_ = 47.09 bar.^17^ Based on the experimental data, the acentric factor can also be easily derived using eq 1b: ω_p_ = 0.3541. Of course, the dependency of the parameters for mixtures on the properties of nanopores has never been explored whatsoever, which also calls for analyses in future investigations.

**Figure 14:**
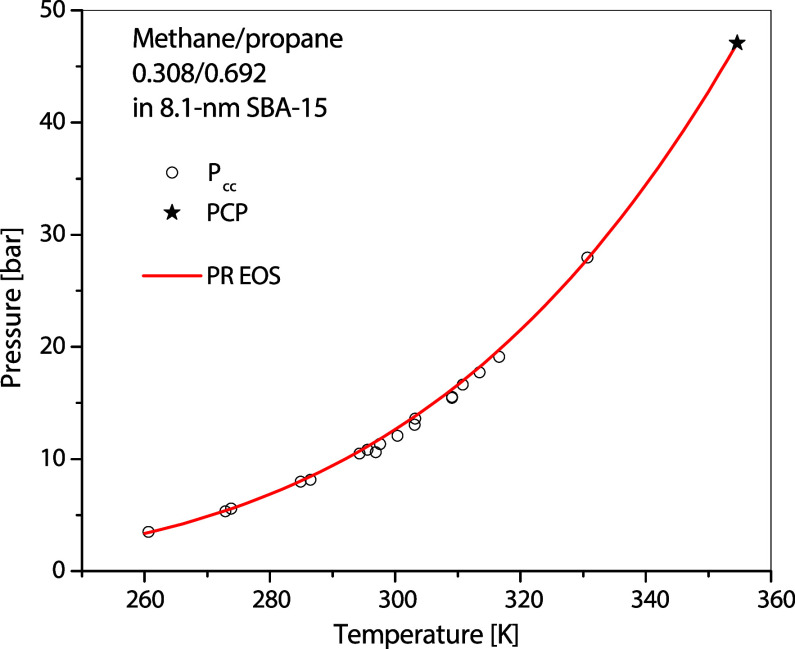
Capillary condensation of methane/propane in 8.1 nm SBA-15 and the calculation using PR EOS based on the measured PCP. Experimental data are taken from Owusu-Banahene et al.^17^ The EOS parameters are listed in Appendix A.

## Conclusion

Two known methods are available in the literature for determining PCP. For the first time, these two methods are implemented on the same confined system for comparison to explore which method provides the true PCP. In this study, both methods are applied to determine the PCP of propane confined in 4.1 nm MCM-41.

The isotherm-based analysis yields a nonzero heat release at its derived PCP-a, suggesting that a phase transition still occurs within the pores. In contrast, the three-line approach identifies its derived PCP-d with zero heat release, indicating that the PCP-d found is the true PCP, beyond which no first-order phase transition occurs. Additionally, the gradual decrease in heat released during capillary condensation with increasing temperature suggests that the first-order phase transition progresses from lower to higher temperatures, passing through the isotherm-based PCP-a before reaching the true critical point at PCP-d.

There is a change in the behavior of condensation in nanopores when the temperature increases, passing through the isotherm-based PCP-a. This change is related to the tensile strength of the condensed liquid phase. At temperatures below this PCP-a, the tensile stress imposed by the pore walls does not surpass the tensile strength of the condensed phase. This means that intermolecular cohesive forces within the fluid exceed the adhesive forces between the fluid molecules and pore walls. Consequently, upon condensation, the resulting condensed phase exhibits a partial wetting behavior. At temperatures above PCP-a, on the contrary, the tension exerted by the pore walls exceeds the tensile strength of the condensed phase. Therefore, the adhesive interaction is stronger than the cohesive interaction, which makes the condensed phase upon condensation spread and completely wet the walls. We now propose to refer to this PCP-a as the pore-wetting transition point (PWTP). Since the concept of critical pore size at constant temperature shares the same underlying physics as PCP for fluid confined in a fixed pore size, the commonly accepted definition of critical pore size corresponding to the pore wetting transition temperature *T*_pw_ at PWTP may need to be reconsidered.

The true PCP of propane in MCM-41 is then applied in EOS to model the system, using the PR EOS. The PR EOS, without modification, successfully reproduces all capillary-condensation curves of confined fluids, both pure fluids with small and large molecules as well as mixtures. These results call for more measurements in the future, as the EOS parameters also depend on the properties of the confining nanopores, such as the pore size and type of material, in addition to the type of fluid. Experimental work on this effort has never been done for confined fluids, despite the same effort being done in the past for the critical points of bulk fluids. Note that the modeling using PR EOS is phenomenological in nature. The pressure of the surrounding bulk fluid, at which the capillary condensation occurs in the pores at a certain temperature, is represented by the saturated vapor pressure of a fictitious bulk fluid having (*T*_Cp_, *P*_Cp_) as the critical point at the same temperature.

## Supplementary Material



## Figures and Tables

**Figure 15:**
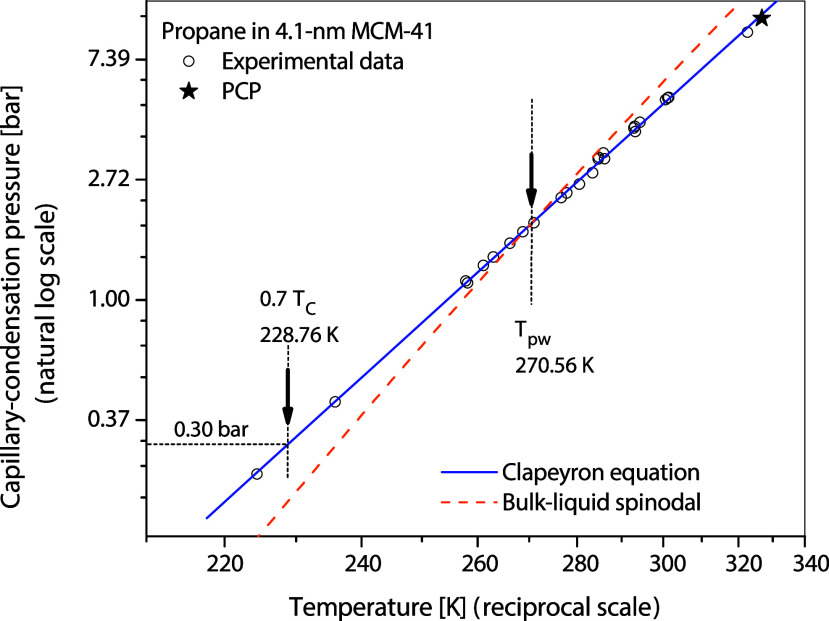
Clapeyron line over all experimental capillary-condensation pressures showing the interpolated condition at 0.7·*T*_Cp_ for ω_p_ calculation, as well as the condition of the intersection with the bulk-liquid spinodal curve to estimate the pore wetting transition point at *T*_pw_.

## Appendix A

The parameters of PR EOS, i.e., the pore critical temperature and pressure (*T*_Cp_, *P*_Cp_), and the acentric factor ω_p_ for confined fluids considered in this work are listed in Table 2.

**Table 2:** Parameters of PR EOS^a^

fluid	nanopores	*T*_Cp_ (K)	*P*_Cp_ (bar)	ω_p_
methane	3.5 nm MCM-41	185.4	24.5	0.1869
	7.0 nm SBA-15	189.5	39.2	0.0926
ethane	3.5 nm MCM-41	275.0	14.3	0.4071
	4.6 nm SBA-15	297.5	34.8	0.2634
	6.1 nm SBA-15	304.1	41.0	0.2171
	8.2 nm SBA-15	304.6	43.2	0.1917
propane	4.1 nm MCM-41	326.8	10.4	0.5398
CO_2_	4.6 nm SBA-15	294.5	51.4	0.3782
	6.1 nm SBA-15	301.3	61.9	0.3327
	8.2 nm SBA-15	303.0	66.4	0.3120
CH_4_/C_3_H_8_	8.1 nm SBA-15	354.6	47.1	0.3541

a Composition: 0.308/0.692 mole fractions.

The acentric factors in Table 2 are calculated using eq 1b. An example of the calculation is that of propane, shown in Figure 15. As the *T*_Cp_ is 326.8 K, *P* ^cc^ is calculated at *T* = 0.7·*T*_Cp_ = 228.76 K. Using the Clapeyron line over all experimental data, which is ln *P* ^cc^ = 10.72344–2728.47112/*T*, *P* ^cc^ at 228.76 K is found to be 0.30 bar, which leads to ω_p_ = 0.5398.

This Clapeyron line intersects the bulk-liquid spinodal, which is calculated using the PC-SAFT EOS in Tan et al.,^25^ at *T*_pw_ = 270.56 K. The corresponding pressure is 1.895 bar. This is the condition of PWTP, where the transition from partial to complete wetting occurs in the nanopores.

## Notes

Experimental data of capillary condensation of propane in MCM-41 using DSC including uncertainty determination, uncertainty determination for PCP, experimental data of capillary condensation of propane in MCM-41 using adsorption/desorption experiments, experimental data of isotherms of propane in MCM-41, and PR-EOS parameter dependency on pore size (PDF)
