# Supplementary material for: Disclosing the True Critical Point of Fluids Confined in Nanopores
Source: Langmuir. 2026 Feb 18;42(8):6191–200. doi: 10.1021/acs.langmuir.5c05643 (PMC12961954; doi:10.1021/acs.langmuir.5c05643)
Supplement: Supplementary file 1 [file la5c05643_si_001.pdf]

Supporting Information

Disclosing the true critical point of fluids confined in nanopores

Ephraim Kakra Owusu-Banahene,[a] Sugata P. Tan,[b] Morteza Dejam[a], and Hertanto Adidharma[c*]
[a]Department of Energy and Petroleum Engineering, University of Wyoming, Laramie, WY 82071, USA
[b]Planetary Science Institute, Tucson, AZ 85719, USA
[c]Department of Chemical and Biomedical Engineering, University of Wyoming, Laramie, WY 82071, USA
*****Email :adidharm@uwyo.edu*

Table of Contents

Experimental data of capillary-condensation of propane in MCM-41 using DSC................................2
The uncertainties of capillary-condensation data of propane in MCM-41 using DSC..........................3
Procedure of uncertainty determination for PCP..4
Capillary condensation data using adsorption/desorption experiments..5
Experimental data of adsorption/desorption isotherms...6
PR-EOS Parameter dependency on pore size..11
References..11

Experimental data of capillary-condensation of propane in MCM-41 using DSC

Table S1. Experimental data of capillary condensation measurements of propane used for PCP determination

12.0 mg MCM-41			13.4 mg MCM-41			20.0 mg MCM-41		
T, K	P, bar	Heat, J	T, K	P, bar	Heat, J	T, K	P, bar	Heat, J
284.7	3.22	0.3956	284.8	3.27	0.5339	285.9	3.39	0.7582
293.1	4.23	0.3056	292.9	4.18	0.4139	294.4	4.38	0.5835
301.4	5.38	0.2227	300.6	5.28	0.317	301.2	5.36	0.4489

Table S2. Experimental data of capillary condensation measurements of propane in MCM-41 from BT 2.15 DSC.

Temperature, K	Pressure, bar
257.8	1.17
261.1	1.33
266.2	1.60
271.0	1.90
277.8	2.43
286.1	3.23

The uncertainties of capillary-condensation data of propane in MCM-41 using DSC

The uncertainties of our measurements are obtained following the procedure documented in Yang et al.[1] The bulk condensation of propane along with the melting point of standard substances from literature are used to derive the uncertainties of measurements in this work. For a Calvet-type DSC, which is used in our work, it has been well documented that the temperature corrections are random in nature.[2] The temperature correction is defined as

$$\Delta T_{corr} = T - T_{exp} \quad \text{(S1)}$$

where T is the temperature obtained from literature ('true' temperature) and T_{exp} is the measured temperature. The mean correction temperature for the micro DSC VII system and the BT 2.15 systems are −0.965 K and 0.480 K, respectively, with corresponding standard deviations of 0.489 K and 0.687 K. These values are obtained by statistically averaging the temperature correction data as shown in Figure 1 and Figure 2. The mean temperature correction is then applied to all temperature measurements in this work, and the standard deviation is the uncertainty of the measurements. The corrected temperature is given by

$$T_{corr} = T_{exp} + \Delta T_{corr,mean} \quad \text{(S2)}$$

with an uncertainty of ± 0.5 K and ± 0.7 K for the micro DSC VII and BT 2.15, respectively.

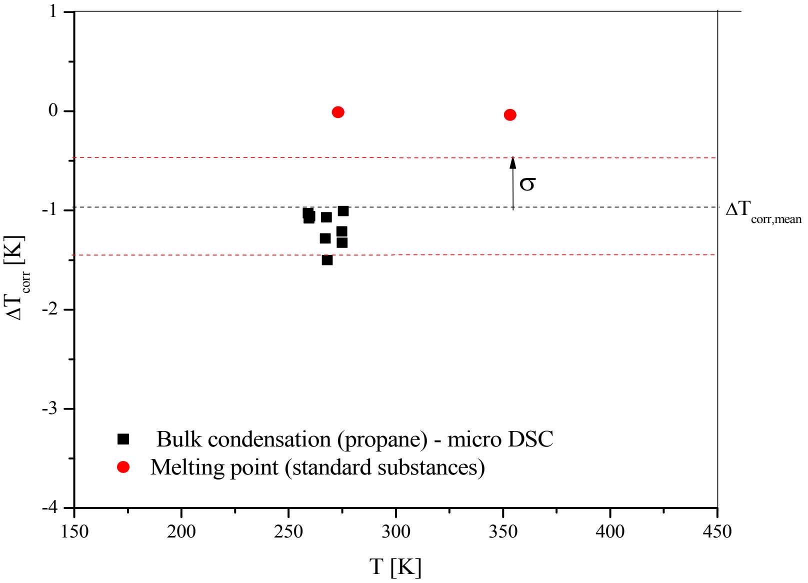

Figure S1. The correction of temperatures for the melting points of standard substances during calibration and bulk propane condensation during capillary condensation measurements using the μDSC VII system.

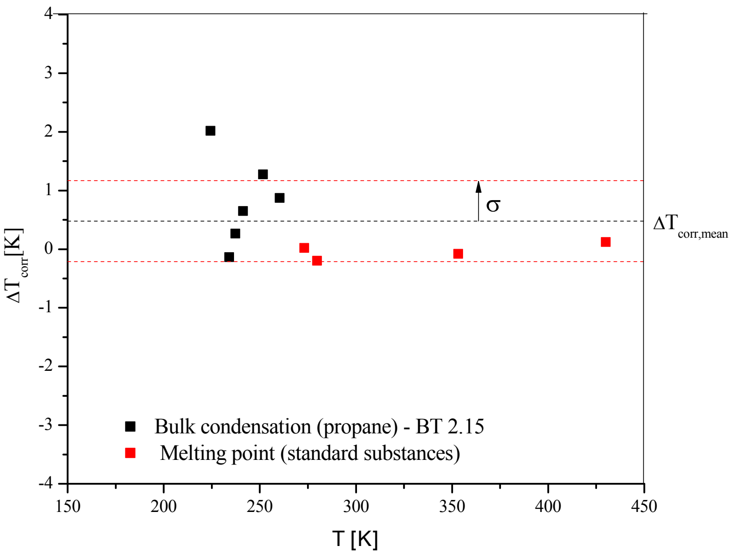

Figure S2. The correction of temperatures for the melting points of standard substances during calibration and bulk propane condensation during capillary condensation measurements using the BT 2.15 system.

Procedure of uncertainty determination for PCP

The procedure to determine the PCP and its uncertainties is the same as that adopted in previous works by our research group.[3,4] The results are given below:

Table S3. Uncertainty determination for T_{Cp} of propane in MCM-41.

Adsorbent amount, mg	Intercept	Standard Error	Intercept + Standard Error	Intercept – Standard Error	Minimum, K	Maximum, K
12.0	0.00307	2.07E-06	3.0721 E-03	3.0679 E-03	325.5	326.0
13.4	0.00306	1.10E-05	3.0710 E-03	3.0490 E-03	325.6	328.0
20.0	0.00306	1.05E-06	3.0611 E-03	3.0590 E-03	326.7	326.9
Mean					325.9	327.0
T_{Cp} derived directly from three-line approach					326.8	
T_{Cp} with uncertainty, K					$326.8^{+0.2}_{-0.9}$	

Table S4. Uncertainty determination for P_{Cp} of propane in MCM-41.

Adsorbent amount, mg	Intercept	Standard Error	Intercept + Standard Error	Intercept – Standard Error	Minimum, bar	Maximum, bar
12.0	2.346	0.01183	2.3578	2.3342	10.32	10.57
13.4	2.355	0.0447	2.3997	2.3103	10.08	11.02
20.0	2.343	0.00589	2.3489	2.3371	10.35	10.47
Mean					10.25	10.69
P_{Cp} derived directly from three-line approach					10.42	
P_{Cp} with uncertainty, K					$10.42^{+0.3}_{-0.2}$	

Capillary condensation data using adsorption/desorption experiments

Table S5. Capillary Condensation Pressures determined from Isotherms.

**Temperature (K)**	**Capillary Condensation Pressure (bar) (Determined from Lorentzian fit of dm/dP against P)**
224.42	0.235
235.87	0.428
258.16	1.150
262.97	1.430
268.77	1.762
276.63	2.339

280.51	2.613
283.46	2.878
293.26	4.054

Experimental data of adsorption/desorption isotherms

Table S6. Experimental data of Adsorption-Desorption Isotherms of Propane in MCM-41.

Temperature = 224.42 K				Temperature = 235.87 K			
Adsorption		Desorption		Adsorption		Desorption	
Pressure, mbar	Adsorption amount, mmol/g	Pressure, mbar	Adsorption amount, mmol/g	Pressure, mbar	Adsorption amount, mmol/g	Pressure, mbar	Adsorption amount, mmol/g
-6.50	.023	699.52	8.580	-6.98	0	900.84	7.960
100.65	2.380	675.30	8.414	99.28	1.598	801.12	7.871
200.11	4.295	625.66	8.247	199.87	2.512	600.19	7.666
210.63	4.624	575.61	8.148	299.50	3.304	475.22	7.450
220.32	5.002	525.34	8.069	310.60	3.411	445.51	6.997
230.52	5.725	475.15	7.994	320.64	3.522	435.29	6.497
240.33	6.699	445.34	7.947	330.71	3.655	425.03	5.933
250.50	7.338	435.18	7.930	340.55	3.818	415.30	5.448
260.51	7.515	425.10	7.913	350.57	4.013	404.91	5.065
271.01	7.570	414.96	7.897	360.63	4.219	394.98	4.818
280.64	7.600	404.76	7.881	370.50	4.413	384.79	4.632
291.09	7.624	394.61	7.866	380.65	4.583	374.74	4.467
300.48	7.647	384.64	7.850	390.61	4.751	364.80	4.288
311.13	7.669	374.85	7.835	400.65	4.973	354.96	4.092
320.70	7.691	364.64	7.819	410.73	5.298	344.90	3.900
330.84	7.710	354.57	7.802	420.50	5.746	334.83	3.729
340.34	7.728	344.80	7.785	430.35	6.312	325.05	3.587
350.50	7.747	334.74	7.764	440.51	6.846	315.15	3.467
360.07	7.765	324.94	7.731	450.02	7.157	304.92	3.360
370.48	7.783	315.23	7.696	500.43	7.516	249.92	2.913
379.99	7.800	305.26	7.667	700.41	7.762	149.89	2.100
390.39	7.818	295.28	7.640	900.84	7.960		
400.00	7.836	285.10	7.613				
410.65	7.852	274.97	7.582				
420.27	7.868	265.04	7.539				
430.69	7.884	255.03	7.428				
440.02	7.899	244.91	6.994				
450.43	7.914	235.02	6.079				
500.70	7.992	224.96	5.221				
550.82	8.068	214.86	4.737				
600.72	8.157	205.02	4.439				

650.49	8.280	149.98	3.086				
699.52	8.580						

Temperature = 258.16 K				Temperature = 262.97 K			
Adsorption		Desorption		Adsorption		Desorption	
Pressure, mbar	Adsorption amount, mmol/g	Pressure, mbar	Adsorption amount, mmol/g	Pressure, mbar	Adsorption amount, mmol/g	Pressure, mbar	Adsorption amount, mmol/g
-6.54	0	2799.92	8.233	-6.94	0	2999.59	7.751
101.56	0.765	2751.30	8.094	99.70	0.655	2902.29	7.704
200.00	1.236	2651.76	7.937	199.98	1.077	2755.09	7.642
300.67	1.651	2551.77	7.851	300.79	1.444	2653.20	7.603
400.06	2.015	2452.22	7.791	400.70	1.768	2552.42	7.566
500.86	2.350	2351.12	7.740	500.80	2.064	2451.75	7.530
601.03	2.663	2250.47	7.694	601.01	2.341	2351.15	7.490
700.83	2.964	2150.88	7.650	700.77	2.602	2249.11	7.450
800.32	3.270	2050.95	7.608	800.77	2.857	2151.70	7.404
900.02	3.630	1951.11	7.562	900.83	3.110	2052.35	7.354
999.71	4.262	1849.79	7.517	1000.55	3.372	1951.43	7.308
1099.99	4.994	1750.16	7.444	1100.49	3.683	1849.61	7.260
1199.91	6.552	1648.82	7.383	1200.61	4.189	1749.37	7.210
1300.37	7.158	1551.42	7.332	1300.45	4.781	1648.52	7.151
1400.39	7.243	1449.57	7.272	1400.27	5.720	1549.68	7.056
1500.37	7.304	1349.74	7.203	1500.07	6.907	1450.18	6.411
1600.80	7.360	1249.81	7.013	1600.43	7.122	1350.24	5.113
1700.34	7.410	1150.40	5.588	1700.96	7.188	1249.89	4.482
1800.12	7.456	1049.67	4.600	1800.71	7.241	1149.82	3.891
1901.03	7.501	949.85	3.893	1899.91	7.289	1049.89	3.506
2000.77	7.544	849.46	3.430	2000.03	7.334	949.82	3.230
2099.94	7.589	750.14	3.111	2099.61	7.377	849.74	2.976
2200.79	7.634	649.31	2.807	2199.45	7.417	749.77	2.724
2300.14	7.678	549.29	2.504	2299.76	7.456	649.60	2.466
2399.58	7.727	449.43	2.187	2401.30	7.493	549.76	2.199
2499.01	7.782	349.48	1.842	2500.13	7.535	449.65	1.915
2599.57	7.854	249.69	1.458	2599.39	7.573	349.56	1.606
2699.53	7.962	149.64	1.019	2699.57	7.612	249.46	1.265
2799.92	8.233			2800.10	7.653	149.59	0.880
				2999.59	7.751		

Table S6. Experimental data of Adsorption-Desorption Isotherms of Propane in MCM-41. (Continued)

Temperature = 268.77 K				Temperature = 276.63 K			
Adsorption		Desorption		Adsorption		Desorption	
Pressure, mbar	Adsorption amount, mmol/g	Pressure, mbar	Adsorption amount, mmol/g	Pressure, mbar	Adsorption amount, mmol/g	Pressure, mbar	Adsorption amount, mmol/g
-6.74	0	3700.78	7.752	-6.45	0	4998.34	7.532
105.36	0.575	3013.84	7.516	501.82	1.362	4754.08	7.365
637.56	2.093	3469.92	7.650	1001.59	2.258	4254.75	7.204
1003.68	2.883	3355.24	7.613	1500.43	3.054	3752.97	7.078
1425.57	3.857	3254.14	7.582	1751.81	3.513	3377.33	6.947
1501.87	4.188	3152.36	7.550	2001.32	4.208	3126.32	6.875
1601.84	4.697	3051.55	7.521	2250.78	5.163	2876.37	6.794
1703.22	5.265	2950.72	7.490	2499.33	6.562	2625.93	6.690
1802.97	6.299	2850.22	7.457	2750.02	6.748	2375.82	6.039
1904.65	6.963	2749.70	7.423	2998.41	6.838	2125.55	4.622
2004.12	7.089	2650.02	7.376	3249.72	6.913	1875.31	3.834
2103.34	7.147	2549.36	7.338	3499.75	6.981	1624.62	3.279
2203.06	7.195	2350.27	7.265	3999.25	7.109	1249.67	2.665
2301.95	7.238	2249.41	7.225	4499.16	7.242	749.33	1.840
2402.40	7.277	1656.55	5.079	4998.34	7.532		
2502.43	7.317	2051.23	7.133				
2701.04	7.385	1949.66	7.057				
2800.93	7.419	1849.77	6.739				
2901.74	7.453	1750.40	5.710				
3002.46	7.485	1649.64	4.936				
3102.64	7.519	1548.73	4.445				
3202.33	7.552	1448.62	3.963				
3302.01	7.586	1198.27	3.309				
3402.27	7.620	798.17	2.480				
3502.18	7.659	297.49	1.250				
3700.78	7.752						

Table S6. Experimental data of Adsorption-Desorption Isotherms of Propane in MCM-41. (Continued)

Temperature = 280.51 K				Temperature = 283.46 K			
Adsorption		Desorption		Adsorption		Desorption	
Pressure, mbar	Adsorption amount, mmol/g	Pressure, mbar	Adsorption amount, mmol/g	Pressure, mbar	Adsorption amount, mmol/g	Pressure, mbar	Adsorption amount, mmol/g
-6.90	.000	5799.28	8.588	-6.42	-1E-4	6009.74	7.3264
500.79	1.228	5656.63	7.573	501.94	1.1367	5756.36	7.224
1001.81	2.051	5257.87	7.275	1001.62	1.908	5257.46	7.098
1500.38	2.761	4881.58	7.165	1500.73	2.5665	4881.35	7.0193
1750.44	3.117	4630.56	7.106	1999.61	3.2109	4632.8	6.9504
2000.14	3.525	4379.77	7.049	2250.15	3.5986	4379.97	6.8843
2250.81	4.111	4129.11	6.955	2500.1	4.148	4127.53	6.8282
2500.97	4.858	3877.26	6.890	2750.78	4.8158	3875.96	6.7681
2751.44	6.181	3626.86	6.827	3000.5	5.9715	3625.62	6.6996
3000.28	6.633	3375.88	6.758	3251.49	6.5621	3375.5	6.6157
3252.59	6.729	3125.77	6.674	3500.56	6.6677	3125.24	6.397
3500.43	6.803	2875.62	6.517	3752.82	6.7424	2875.13	5.3013
3750.47	6.868	2624.63	5.416	4000.28	6.8061	2624.64	4.4594
4000.23	6.927	2374.60	4.443	4250.98	6.8621	2374.27	3.847
4250.52	6.983	2124.75	3.778	4500.27	6.9163	2124.35	3.3934
4500.61	7.042	1874.45	3.301	4750.01	6.9685	1749.76	2.8863
4750.88	7.096	1624.56	2.931	4999.88	7.0225	1249.55	2.2493
5000.14	7.157	1248.88	2.409	5503.94	7.1326	749.23	1.5466
5498.75	7.352	748.70	1.661	6009.74	7.3264		
5799.28	8.588						

Table S6. Experimental data of Adsorption-Desorption Isotherms of Propane in MCM-41. (Continued)

Temperature = 293.26 K				Temperature = 249.53 K			
Adsorption		Desorption		Adsorption		Desorption	
Pressure, mbar	Adsorption amount, mmol/g	Pressure, mbar	Adsorption amount, mmol/g	Pressure, mbar	Adsorption amount, mmol/g	Pressure, mbar	Adsorption amount, mmol/g
-6.76	0	7508.90	6.997	-6.71	0	1800.08	7.907
503.76	.885	7263.39	6.951	99.55	0.998	1751.20	7.874
1003.93	1.508	6895.10	6.889	199.97	1.617	1651.52	7.815
1523.98	2.060	6639.70	6.849	300.3	2.134	1551.91	7.758
2027.06	2.545	6386.98	6.808	400.64	2.589	1450.95	7.700
2555.39	3.046	6136.89	6.755	500.2	3.014	1350.35	7.640
3037.47	3.563	5883.51	6.713	600.57	3.468	1249.98	7.581
3562.05	4.404	5630.61	6.669	700.49	4.328	1149.70	7.502
4011.97	5.526	5378.31	6.624	800.13	5.690	1049.80	7.425
4255.67	6.190	5129.28	6.574	900.25	7.269	949.85	7.340
4502.95	6.404	4880.97	6.516	1000.34	7.382	849.88	6.958
4749.62	6.486	4631.62	6.448	1100.21	7.461	750.23	4.830
4999.80	6.548	4379.88	6.332	1200.79	7.530	649.91	3.825
5252.15	6.602	4129.42	5.899	1300.6	7.593	549.78	3.237
5498.03	6.650	3753.60	4.789	1400.4	7.652	449.69	2.809
5749.52	6.694	3251.72	3.869	1500.28	7.714	349.55	2.374
6001.42	6.736	2750.55	3.247	1600.3	7.774	249.69	1.897
6253.18	6.776	2251.30	2.762	1700.14	7.837	149.69	1.341
6497.38	6.818	1751.05	2.288	1800.08	7.907		
6749.75	6.857	1249.75	1.783				
6997.54	6.897	749.59	1.213				
7508.90	6.997						

PR-EOS Parameter dependency on pore size

The success of the PR EOS application intrigues further inquiry on the dependency of the parameters on the properties of the nanopores where the fluids are confined in, at least the pore size. As this is beyond the purpose of this work, we only show the data plots in Figure 3 as an initial rough illustration of some interesting work awaiting ahead.

The LJ diameters σ are taken from Hirschfelder, et al.:[5] 0.3817 nm for methane, 0.3954 nm for ethane, 0.5637 nm for propane, and 0.407 nm for CO_2. The parameters show some trends in the plots for future investigations to work on.

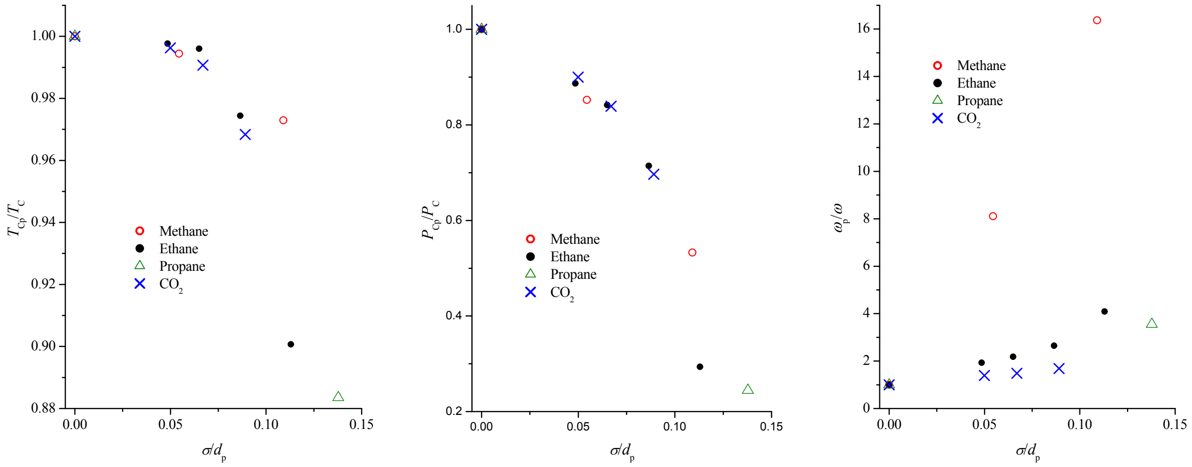

Figure S3. Plots of the PR EOS parameters against the ratio between molecular LJ diameters σ and the pore diameter d_p (see Appendix A and Table A1). T_C, P_C, and ω are critical temperature, pressure, and the acentric factor of the bulk fluids.[6]

References

1. Qiu, X.; Tan, S.P.; Dejam, M.; Adidharma, H. Experimental Study on the Criticality of a Methane/Ethane Mixture Confined in Nanoporous Media. *Langmuir*. **2019**;35(36):11635-11642. doi:10.1021/acs.langmuir.9b01399
2. De Castro, C.A.N.; Lourenço, M.J.V.; Sampaio, M,O. Calibration of a DSC: its importance for the traceability and uncertainty of thermal measurements. *Thermochim. Acta* **2000**;347(1-2):85-91. doi:10.1016/S0040-6031(99)00420-7
3. Yang, H.; Jayaatmaja, K.; Dejam, M.; Tan, S.P.; Adidharma, H. Phase Transition and Criticality of Methane Confined in Nanopores. *Langmuir*.**2022**;38(6):2046-2054. doi:10.1021/acs.langmuir.1c02955
4. Yang, H.; Dejam, M.; Tan, S.P.; Adidharma, H. First-order and gradual phase transitions of ethane confined in MCM-41. *Phys. Chem. Chem. Phys*.**2022**:18161-18168. doi:10.1039/d2cp02530b
5. Hirschfelder, J. O.; Curtiss, C. F.; Bird, R. B. *Molecular Theory of Gases and Liquids*; John Wiley & Sons, Inc.: New York, 1964.
6. Yang, X.; Richter, M. Effective Thermophysical Constants of Thousands of Fluids. I: Critical Temperature, Critical Pressure, Critical Density, and Acentric Factor. *J. Chem. Eng. Data* **2025**, 70, 2911-2946. doi: 10.1021/acs.jced.5c00110.
